# Long-Term Effects of Traffic-Related Air Pollution on Mortality in a Dutch Cohort (NLCS-AIR Study)

**DOI:** 10.1289/ehp.10767

**Published:** 2007-11-19

**Authors:** Rob Beelen, Gerard Hoek, Piet A. van den Brandt, R. Alexandra Goldbohm, Paul Fischer, Leo J. Schouten, Michael Jerrett, Edward Hughes, Ben Armstrong, Bert Brunekreef

**Affiliations:** 1 Institute for Risk Assessment Sciences, Division Environmental Epidemiology, Utrecht University, Utrecht, the Netherlands; 2 Department of Epidemiology, Maastricht University, Maastricht, the Netherlands; 3 TNO Quality of Life, Department of Prevention and Health, Leiden, the Netherlands; 4 Centre for Environmental Health Research, National Institute for Public Health and the Environment (RIVM), Bilthoven, the Netherlands; 5 School of Public Health, University of California, Berkeley, California, USA; 6 Edward Hughes Consulting, Ottawa, Ontario, Canada; 7 Public and Environmental Research Unit, London School of Hygiene and Tropical Medicine, London, United Kingdom; 8 Julius Center for Health Sciences and Primary Care, University Medical Center Utrecht, Utrecht, the Netherlands

**Keywords:** air pollution, cohort, mortality, traffic

## Abstract

**Background:**

Several studies have found an effect on mortality of between-city contrasts in long-term exposure to air pollution. The effect of within-city contrasts is still poorly understood.

**Objectives:**

We studied the association between long-term exposure to traffic-related air pollution and mortality in a Dutch cohort.

**Methods:**

We used data from an ongoing cohort study on diet and cancer with 120,852 subjects who were followed from 1987 to 1996. Exposure to black smoke (BS), nitrogen dioxide, sulfur dioxide, and particulate matter ≤mu;M_2.5_), as well as various exposure variables related to traffic, were estimated at the home address. We conducted Cox analyses in the full cohort adjusting for age, sex, smoking, and area-level socioeconomic status.

**Results:**

Traffic intensity on the nearest road was independently associated with mortality. Relative risks (95% confidence intervals) for a 10-μg/m^3^ increase in BS concentrations (difference between 5th and 95th percentile) were 1.05 (1.00–1.11) for natural cause, 1.04 (0.95–1.13) for cardiovascular, 1.22 (0.99–1.50) for respiratory, 1.03 (0.88–1.20) for lung cancer, and 1.04 (0.97–1.12) for mortality other than cardiovascular, respiratory, or lung cancer. Results were similar for NO_2_ and PM_2.5_, but no associations were found for SO_2_.

**Conclusions:**

Traffic-related air pollution and several traffic exposure variables were associated with mortality in the full cohort. Relative risks were generally small. Associations between natural-cause and respiratory mortality were statistically significant for NO_2_ and BS. These results add to the evidence that long-term exposure to ambient air pollution is associated with increased mortality.

Although air pollution concentrations have decreased substantially over the last several decades, recent studies from the United States found associations between long-term exposure to air pollution and cardiopulmonary and lung cancer mortality (Abbey et al. 1999; Dockery et al. 1993; Jerrett et al. 2005; Laden et al. 2006; Miller et al. 2007; Pope et al. 1995, 2002). Cohort studies from Europe have tended to confirm the U.S. findings (Filleul et al. 2005; Gehring et al. 2006; Hoek et al. 2002; Nafstad et al. 2004), but the emphasis has been on different pollutants and on different exposure assessment methods. The U.S. studies have used data from single monitoring stations to characterize exposure of subjects living in that city, or spatial interpolation from multiple monitoring stations. Most European studies have estimated exposure at the home address using dispersion or stochastic modeling and variables such as living close to busy roads. In a previous Dutch study in 5,000 subjects, a random sample from a full cohort (*n* ∼ 120,000), cardiopulmonary and all-cause mortality were associated with living close to a major road, with relative risks of 1.95 [95% confidence interval (CI), 1.09–3.52] and 1.41 (95% CI, 0.94–2.12), respectively (Hoek et al. 2002). In this article we extend this work to the full cohort with a much larger number of deaths and with an improved exposure assessment method.

## Materials and Methods

### Study design

The cohort has been described in detail elsewhere (van den Brandt et al. 1990a). Briefly, the Netherlands Cohort Study on Diet and Cancer (NLCS) was initiated in September 1986 with the enrollment of 120,852 subjects (58,279 males and 62,573 females) 55–69 years of age living in 204 municipalities located throughout the country. The study was designed as a case–cohort study: Cases are derived from the entire cohort, whereas the person-years at risk are estimated from a random subcohort (*n* = ∼5,000) (Volovics and van den Brandt 1997). This approach was selected for efficiency of baseline questionnaire processing and avoidance of active follow-up of the entire cohort. At baseline, all participants completed an 11-page questionnaire on dietary habits and other risk factors for cancer. For all participants, data from one machine-readable page of the questionnaire were entered at baseline (with information about age, sex, and smoking status). After recruitment, the entire cohort was followed up for cancer incidence by record linkage to cancer registries (van den Brandt et al. 1990b). For the emerging cases and the randomly selected subcohort, the remaining 10 questionnaire pages (not machine readable) were manually entered, blinded with respect to case–subcohort status. The exact residential address at baseline was available for all study participants.

Mortality was assessed between 1 January 1987 and 31 December 1996. Mortality data were obtained from the Dutch Central Bureau of Genealogy and the Dutch Central Bureau of Statistics (unpublished data). The cause of death was coded according to the *International Classification of Diseases, 9th Revision* [ICD-9; World Health Organization (WHO) 1975] (for 1986–1995) and *10th Revision* (ICD-10; WHO 1993) (for 1996). Causes of death were grouped into natural cause, cardiopulmonary, cardiovascular, respiratory, lung cancer, and mortality other than cardiopulmonary or lung cancer (Table 1). The NLCS study was approved by institutional review boards from Maastricht University and the Netherlands Organization for Applied Scientific Research. All cohort members consented to participation by completing a mailed, self-administered questionnaire.

### Air pollution exposure assessment

Details of the exposure assessment method have been described previously (Beelen et al. 2007). In summary, long-term exposure to outdoor air pollution at the 1986 home address was estimated for all participants as the sum of regional, urban, and local traffic contributions. The home addresses were geocoded into standard Dutch geographic coordinates [Address Coordinates Netherlands (ACN)] using a database from 2000 consisting of all registered addresses by the Dutch postal service (unpublished data). The accuracy of ACN is high, with 93.5% of all coordinates located at the centroid of the correct building, 6.0% located at the centroid of the correct parcel, and only 0.5% not located in the correct building or parcel (Kadata 2001). No information was available about the exact work addresses of participants.

We estimated regional background concentrations using inverse distance weighed interpolation of concentrations measured at regional background sites in the National Air Quality Monitoring Network (NAQMN). We estimated the additional urban component using regression models with residual concentrations for all regional background and urban monitoring sites in the NAQMN as dependent variable, calculated as measured concentration minus estimated regional component concentration using cross-validation. As predictor variables, we used the number of inhabitants around a monitoring site and land-use variables that indicated whether a site was located in a city center, in a rural background location, or in an industrial location. The sum of the regional and urban contributions was defined as background concentration. Background concentrations were estimated for nitrogen dioxide, black smoke (BS), and sulfur dioxide. Average concentrations were estimated for 1976–1985 and 1987–1996 (in 1986 the NAQMN was rearranged, resulting in only limited days with valid measurements in 1986). We estimated the background concentration for fine particles < 2.5 μm in diameter (PM_2.5_) by converting PM_10_ (particles < 10 μm) concentrations, measured in the NAQMN from 1992 to 1996, into PM_2.5_ concentrations using a single ratio, established from monitoring data in the Netherlands. This was done because PM_2.5_ was not monitored in the Netherlands during the study period.

Local traffic contributions were characterized by traffic variables that we estimated using a geographic information system (GIS) from a digital road network to which traffic intensity data (average total number of motor vehicles per 24 hr (mvh/24hr), including weekdays and weekend) from 1986 were linked. Because traffic intensity data were available for different years for municipal roads, we extrapolated traffic intensities to 1986 for roads for which 1986 traffic data were not available. Extrapolation was based on trends estimated from traffic intensity data from municipalities with multiple years of data (Beelen et al. 2007). Traffic intensities were linked to the National Road Database [Nationaal WegenBestand (NWB); unpublished data], based on road name and/or road number. The NWB includes all roads in the Netherlands that have a street name and/or road number. More than 98% of the Dutch roads have been included. More than 95% of all road sections in the NWB have a maximum location difference of 10 m compared with the true location (Beelen et al. 2007). The digital road network and the coordinate database for geocoding addresses were from the same time period, and both used the same standard Dutch coordinate system; therefore, substantial error due to geographic differences between the two databases is unlikely.

We used the following as traffic variables: *a*) traffic intensity on nearest road; *b*) sum of traffic intensity in a 100-m buffer around each residential address; *c*) traffic intensity on the nearest major road (with > 10,000 mvh/24hr) and distance to this road; and *d*) an indicator variable “living within 100 m of a motorway and/or within 50 m of a local road with traffic intensity > 10,000 mvh/24hr.” Further, we obtained quantitative estimates for the local component for NO_2_, BS, and PM_2.5_ from field-monitoring campaigns (Beelen et al. 2007). We estimated no local traffic contribution for SO_2_ because there is virtually no traffic contribution to this pollutant. These local component concentrations were added to the background concentrations, producing an overall exposure estimate for each pollutant.

### Statistical analysis

We analyzed air pollution effects for overall concentrations and for a combination of background concentrations and traffic variables to identify effects of living near busy roads separately.

We calculated relative risks (RRs) for concentration and traffic variable differences between the 5th and the 95th percentiles of the distributions. For NO_2_ this was rounded to 30 μg/m^3^, for BS 10 μg/m^3^, for SO_2_ 20 μg/m^3^, and for PM_2.5_ 10 μg/m^3^. For the traffic variables the differences between the 5th and the 95th percentile were 10,000 mvh/24hr for the traffic intensity on the nearest road, 335,000 mvh/100m for the sum of traffic intensity in a buffer of 100 m, 20,000 mvh/24hr for the traffic intensity on the nearest major road, and 2.3 m for the natural logarithm of distance to this road.

We conducted analyses in the full cohort using Cox proportional hazards models. Person-years were calculated for all participants from baseline until death or end of follow-up. Person-years for subjects who died from causes other than those being analyzed were judged censored at time of death in cause-specific analyses.

We adjusted for sex, age at baseline, and smoking status coded as never, ex, and current smoker separately for cigarette, cigar, and pipe smoking. We further adjusted for area-level indicators assessed using GIS data from the Central Bureau of Statistics (CBS; unpublished data): percentage of persons with a low and with a high income at the neighborhood scale and the COROP area scale. COROP areas have been defined in 1970 by the Coordination Commission for Regional Research Program such that each COROP area consists of a central point (e.g., a city) and the surrounding economic and social region. The Netherlands is divided in 40 COROP areas. We chose two scales for area-level socioeconomic status because life expectancy varies significantly by COROP area (de Hollander et al. 2006), and the neighborhood scale does not capture such regional variations adequately. Low income was defined by the CBS as below the 40th percentile and high income as above the 80th percentile of the Dutch income distribution (Table 2).

We used Cox-Poisson random effects survival software as described by Jerrett et al. (2005) to incorporate spatial clustering at the municipal and/or neighborhood scale in the full cohort analyses. Both one-level (i.e., municipality or neighborhood) and two-level (i.e., municipality and neighborhood) analyses were conducted, using independent-clusters and distance–decay random-effects models.

We assessed effect modification by sex and cigarette-smoking status in the full cohort by conducting separate analyses in subgroups. We also conducted separate subgroup analyses for the percentage of persons with low income in a neighborhood and COROP area (in tertiles).

We tested heterogeneity in pollution relative risks across estimates from different subgroups using Cochran’s Q test (DerSimonian and Laird 1986).

We also conducted case–cohort analyses, including only subcohort members and cases (deaths), which is the standard data analysis approach in the NLCS. Cases were enumerated from the entire cohort, whereas person-years for the entire cohort were estimated using the random subcohort of 4,971 participants. We analyzed data with Cox proportional hazards models. To account for additional variance introduced by sampling from the cohort, we estimated standard errors using the robust Huber–White sandwich estimator (Lin and Wei 1989).

In the case–cohort analyses we adjusted for variables chosen *a priori*: sex; age at baseline; active cigarette, cigar, and pipe smoking coded as never/ex/current and number of cigarettes/cigars/pipes and number of years of smoking; passive smoking defined as whether the partner smoked; educational level in three categories: primary school, lower vocational education, and high school and higher; the last occupation of the participant was coded in six categories: never paid work, blue collar, lower white collar, upper white collar, other, and whether the last occupation was > 40 years ago; occupational exposure of the last occupation to biological dust, mineral dust, and gases and fumes coded as no, low, and high exposure assessed using the community-based ALOHA job exposure matrix (Sunyer et al. 1998); marital status in two categories: married, and never married, divorced, or widowed; body mass index (BMI) in categories < 20, 20–25, 25–30, and > 30 kg/m^2^; alcohol consumption in five categories: none, 0–5, 5–15, 15–30, and > 30 g/day; dietary habits as continuous variables: intake of vegetables, fruit, fish, and energy-adjusted intake of fiber, folate, and saturated, monounsaturated, polyunsaturated, and trans fat; and for the area-level indicators of socioeconomic status.

No methods were available for spatial autocorrelation analyses in a case–cohort setting. We assessed effect modification by educational level, fruit consumption (in tertiles), and vegetable consumption (in tertiles) in the case–cohort by conducting separate analyses in subgroups.

Information about moving during the follow-up period was available only in the case–cohort group. We conducted a separate analysis for subjects who did not move during the follow-up period. We also conducted separate analyses in the case–cohort data set for people who had a paid job and who did not have a paid job at baseline.

Data management was done using SPSS 12.0 (SPSS Inc., Chicago, USA), and statistical analyses were conducted using STATA statistical software, version 8 (StataCorp, College Station, TX, USA). GIS calculations were conducted using ArcInfo (ESRI, Redlands, WA, USA). Spatial analyses were conducted using the R interface to the software described by Ma et al. (2003).

## Results

We identified a geographic coordinate for the home address for 97% of the subjects (*n* = 117,528). About 15% of the subjects died during follow-up (Table 1). Population characteristics of subjects for whom a coordinate was available are summarized in Table 2.

### Exposure data

**Figure 1:**
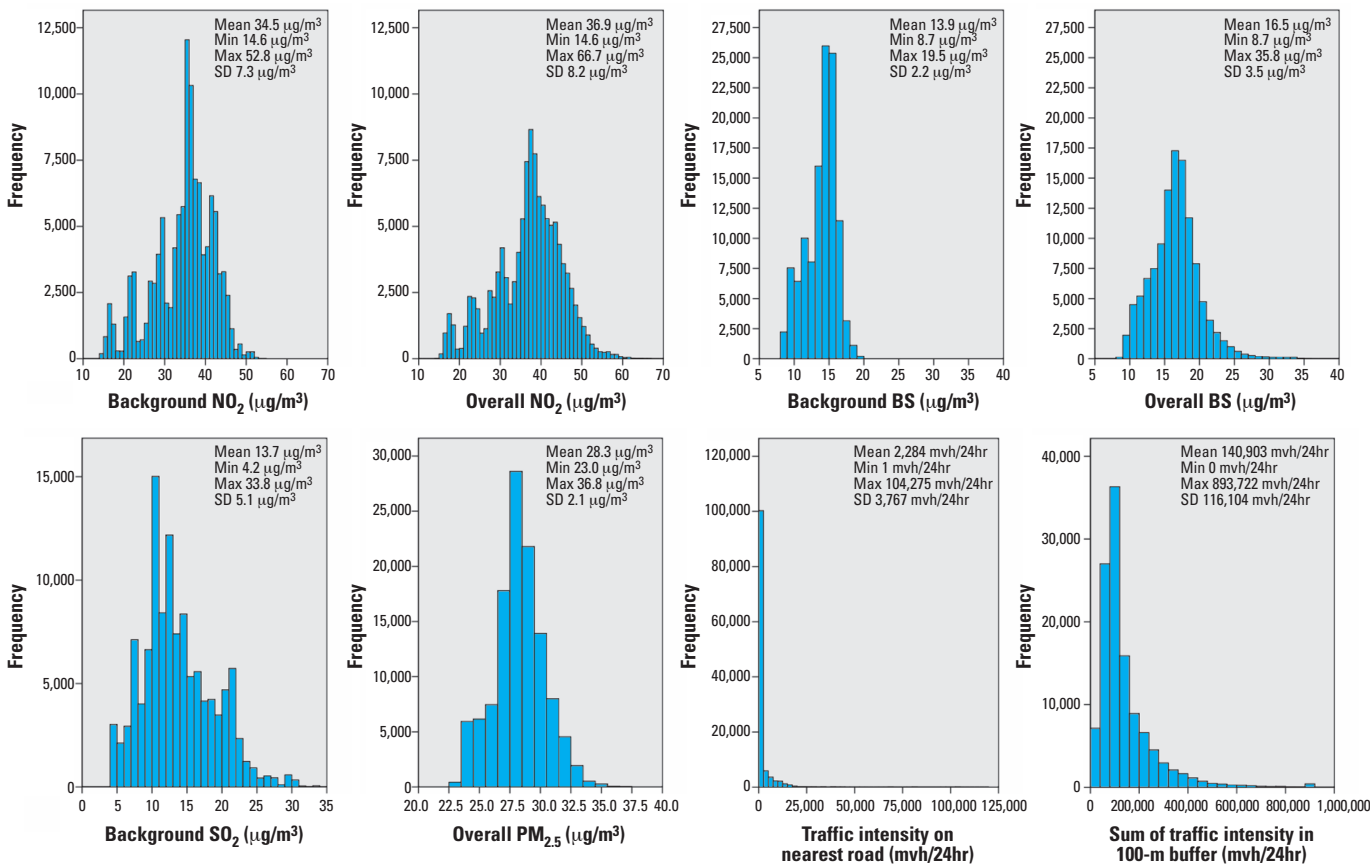
Distribution of estimated NO_2_ (background and overall estimate), BS (background and overall estimate), SO_2_ (background), and PM_2.5_ (overall estimate) concentrations (1987–1996), and of the traffic intensity on the nearest road and the sum of traffic intensity in a 100-m buffer, at the 1986 home address (*n* = 117,528). Abbreviations: Max, maximum; mi, minimum.

Background concentrations estimated for the periods 1976–1985 and 1987–1996 were highly correlated (> 0.9) for each pollutant. The correlations between different air pollutants within the same period were all > 0.8, except for SO_2_ (> 0.6) (Beelen et al. 2007). Figure 1 illustrates that there is considerable contrast of exposure in the full cohort: 5,784 participants (4.9%) lived within 50 m of a road > 10,000 mvh/24hr and/or within 100 m of a motorway; 4.4% lived within 50 m of a road > 10,000 mvh/24hr, and 0.6% lived within 100 m of a motorway. Correlations between background BS and traffic intensity on the nearest road and sum of traffic intensity in a 100 m buffer were modest: 0.12 and 0.28, respectively.

### Associations between mortality and air pollution concentrations

Table 3 shows the associations between overall air pollution concentrations and mortality in the full cohort and case–cohort analyses.

In the full cohort analyses, there was no association between estimated SO_2_ and mortality in any of the analyses. BS and NO_2_ were significantly associated with natural-cause and respiratory mortality. Effect estimates for other mortality were also increased with RRs similar to the effect estimates for natural-cause mortality. The effect estimate in the full cohort for cardiopulmonary mortality was 1.07 (95% CI, 0.98–1.15) for BS for a 10-μg/m^3^ increase in concentration.

In the adjusted case–cohort analyses, there was no association between air pollution and mortality.

Further analyses showed that age–sex adjusted results between case–cohort and full cohort analyses were comparable, especially for BS and NO_2_, but adjusted results were not. This was related to the loss of about 40% of subjects in the adjusted case–cohort analyses because of missing values in one or more confounder variables. Further analyses in the case–cohort sample showed little difference between the effect estimates adjusted for all available confounders and adjusted for only the limited set of confounders available in the full cohort, when the analysis was restricted to subjects without missing values. Because of these issues with the case–cohort analysis and evidence that residual confounding in the full cohort is unlikely to be substantial, here we focus mainly on the full cohort results. Appendix 1 of the Supplemental Material (online at http://www.ehponline.org/members/2007/10767/suppl.pdf) provides a more detailed discussion of these differences between case–cohort and full cohort results. Case–cohort analyses were, of course, also less precise, especially for the more frequent outcomes for which the ratio between subcohort size and number of cases was smaller.

### Associations between mortality and traffic variables

Table 4 shows the adjusted associations between the traffic variables and cause-specific mortality in the full cohort and case–cohort analyses. Effect estimates for traffic variables were independent of background pollutant in the model, so we present relative risk estimates for traffic variables from models with BS background concentrations (1987–1996). Effect estimates were not sensitive to different confounder models for both full cohort and case–cohort analyses (data not shown).

In the full cohort analyses, risks for all traffic variables were elevated for all mortality outcomes, except for other mortality. The relative risk estimate for the association between cardiopulmonary mortality and traffic intensity on nearest road was 1.06 (95% CI, 1.00–1.12) for an increase of 10,000 mvh/24hr. No association was found with any of the mortality outcomes for traffic intensity on the nearest major road and distance to this road (data not shown).

The adjusted case–cohort analysis did not show any association with traffic variables. However, further analysis showed that this was not attributed to the additional adjustment (for the case–cohort analyses a larger number of confounders was available compared with the full cohort analyses), but because sampling the subcohort introduced random error in a downward direction, probably related to the small fraction of high exposed subjects and the skewness of the exposure distribution of the traffic variables (Figure 1). Appendix 1 of the Supplemental Material (online at http://www.ehponline.org/members/2007/10767/suppl.pdf) provides a more detailed discussion of the effect of random variability in the case–cohort analyses.

### Spatial analyses, effect modification, and moving

**Figure 2:**
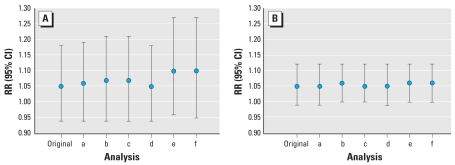
Adjusted results of spatial analyses for association between cardiopulmonary mortality and BS background concentration (1987–1996) (*A*) and traffic intensity on the nearest road in the full cohort (*n* = 107,005) (*B*). RRs and 95% CIs are shown for the original, 1-level neighborhood independent-clusters (analysis a), 1-level municipality independent-clusters (analysis b), 2-level independent-clusters (analysis c), 1-level neighborhood distance-decay (analysis d), 1-level municipality distance-decay (analysis e), and 2-level distance-decay (analysis f) analyses (confounders used are age, sex, smoking status, and area-level indicators of socioeconomic status).

When spatial autocorrelation was taken into account, full cohort results did not change appreciably (Figure 2).

**Figure 3:**
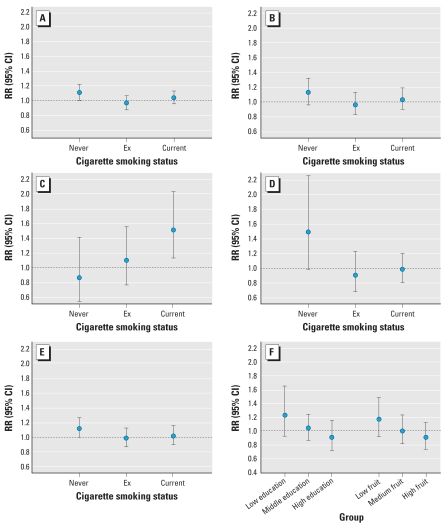
Association between black smoke overall concentration (1987–1996) and cause-specific mortality in subgroups for cigarette smoking status in the full cohort data set (*A–E*), and (*F*) by education and fruit consumption in the case–cohort data set. (*A*) Natural-cause (*p* = 0.15), (*B*) cardiovascular (*p* > 0.2), (*C*) respiratory (*p* = 0.11), (*D*) lung cancer (*p* = 0.14), and (*E*) other mortality (*p* > 0.2). (*F*) Education of the household coded as low = only primary school; middle = lower vocational education; and high = junior high school, senior high school, higher vocational education, and university (*p* > 0.2). Fruit consumption divided in tertiles: low, 0–96.8 g/day; medium, 96.8–191.8 g/day; and high, > 191.8 g/day. Adjusted for age, sex, smoking status, and area-level indicators of socioeconomic status (*p* > 0.2). *p*-Value, Cochran’s Q test for heterogeneity.

In the full cohort analyses, there were no significant differences in effect estimates between men and women. The effect estimates for respiratory mortality were higher among current smokers, whereas there was suggestive evidence that effect estimates for natural-cause and lung cancer mortality were higher among never smokers (Figure 3A, D). Air pollution effect estimates for natural-cause mortality were significantly higher for people living in neighborhoods with the lowest percentage of persons with a low income. Differences between subgroups were, however, inconsistent for the different causes of death; for example, for cardiovascular mortality the lowest and medium tertile had the same relative risk, and for respiratory mortality the highest risk was found in the subgroup with the highest percentage of low-income subjects. Subgroup analyses in the case–cohort data set were adjusted for the limited number of confounders available for the full cohort to avoid the described selection effect that is present when the case–cohort analyses are adjusted for all available confounders. Effect estimates for the full case–cohort data set adjusted for the limited confounder model used in the full cohort analyses were for BS overall concentrations 1.03 (95% CI, 0.91–1.17) for natural-cause mortality, 1.02 (95% CI, 0.88–1.18) for cardiovascular mortality, 1.16 (95% CI, 0.91–1.48) for respiratory mortality, 0.99 (95% CI, 0.80–1.23) for lung cancer mortality, and 1.03 (95% CI, 0.91–1.16) for other mortality. Figure 3F shows that the effect estimates for natural-cause mortality for BS were higher in those with low education and in those with low fruit consumption in the case–cohort analyses. Relative risks for the different groups, however, did not differ significantly. Trends were similar for cardiovascular and other mortality (data not shown). Effects estimates for the different mortality outcomes for BS overall concentrations were not different for different tertiles of vegetable consumption (data not shown).

Approximately 30% of the participants moved residence between 1986 and end of follow-up (Beelen et al. 2007). Effect estimates from case–cohort analyses for the association between air pollution and mortality were higher for subjects who did not move during the follow-up period compared with all subjects, though not significantly so. Effect estimates for subjects who did not move during the follow-up period were for BS overall concentrations 1.13 (95% CI, 0.97–1.31) for natural-cause mortality, 1.12 (95% CI, 0.94–1.34) for cardiovascular mortality, 1.39 (95% CI, 1.01–1.90) for respiratory mortality, 1.14 (95% CI, 0.88–1.48) for lung cancer mortality, and 1.10 (95% CI, 0.94–1.28) for other mortality.

At baseline approximately 85% of the participants had no paid job. Effect estimates of case–cohort analyses for natural-cause mortality were not different for participants who had no paid job at baseline (1.05; 95% CI, 0.91–1.21) and for participants who had a paid job at baseline (0.97; 95% CI, 0.74–1.29), and did not differ with the results for the entire case–cohort sample. Results for the other mortality outcomes were similar.

## Discussion

In the full cohort, traffic-related air pollution and several traffic exposure variables were associated with mortality. Relative risks were generally small. Statistically significant associations between NO_2_ and BS exposure and natural-cause and respiratory mortality were found. The highest relative risks were found for respiratory mortality. Relative risks were also elevated but not significant for mortality other than cardiovascular, respiratory, or lung cancer mortality.

For the first time in Europe, we now report a relative risk estimate for PM_2.5_ based on PM_2.5_ derived from monitored PM concentrations. Effect estimates for PM_2.5_ for the full cohort analyses, although not statistically significant, were quantitatively comparable for natural-cause mortality to those of the American Cancer Society (ACS) study in the United States (Pope et al. 2002). In the ACS study, strongest associations were found between cardiovascular mortality and fine particulate exposure, whereas we found only slightly, not significantly elevated risks for PM_2.5_ exposure and this subcategory of mortality. The ACS study (Pope et al. 2004) and the extended follow-up of the Six Cities Study (Laden et al. 2006) were unable to address traffic intensity variables for which we found some associations with especially respiratory mortality.

Our findings are coherent with other cohort studies that reported significant associations between respiratory mortality and long-term exposure to air pollution (Abbey et al. 1999; Nafstad et al. 2004). Numerous studies have also found effects of long-term and short-term exposure to air pollution on respiratory morbidity (Brunekreef and Holgate 2002; Gauderman et al. 2007). Further, time-series studies have found associations between short-term changes in air pollution and both cardiovascular and respiratory mortality.

We also found nonsignificantly elevated risks for the association between mortality other than cardiopulmonary or lung cancer mortality and overall air pollution. This mortality outcome, however, includes several cancer types and other mortality outcomes that might also be associated with air pollution.

A study in a subpopulation of the ACS study in Los Angeles, California, estimated exposure to PM_2.5_ at an intraurban scale (Jerrett et al. 2005). Relative risks associated with a 10-μg/m^3^ increase of PM_2.5_ concentrations were 1.17 (95% CI, 1.05–1.30) for all-cause, 1.12 (95% CI, 0.97–1.30) for cardiopulmonary, and 1.44 (95% CI, 0.98–2.11) for lung cancer mortality. The results suggested that the health effects associated with within-city gradients in PM_2.5_ concentrations may be larger than previously found across metropolitan areas (Jerrett et al. 2005). A recent large cohort study on long-term exposure to fine particulate air pollution and cardiovascular events among post-menopausal women in 36 U.S. metropolitan areas also found that effect estimates within cities were larger than effect estimates between cities. The risk of cardiovascular death with higher levels of PM_2.5_ was larger than the estimates reported in the previous U.S. cohort studies. A relative risk of 1.76 (95% CI, 1.25–2.47) was found for cardiovascular mortality for an increase of 10 μg/m^3^ in PM_2.5_ concentrations (Miller et al. 2007). In the current study we assessed air pollution on an even finer spatial scale than the Jerrett et al. (2005) and Miller et al. (2007) studies, because we took traffic near the home into account, but we nevertheless found RR estimates for PM_2.5_ that were more comparable to the national ACS study, which assessed between-city variability of concentrations.

In the present study, we refined the traffic exposure variables by using a more precise road network and adding actual traffic intensity data to the network compared with the previous study (Hoek et al. 2002). With these more refined traffic variables, we did observe associations with cause-specific mortality, but the effect estimates were smaller than reported previously for the less refined exposure indicator in a random subgroup of the full cohort. This is related partly to sampling from the full cohort and to the difference in follow-up: Adding an extra 2 years of follow-up reduced the relative risk for living near a major road in the subcohort from 1.95 to 1.34. The previous study results were based on about only 500 deaths and a small number of study subjects producing wide confidence intervals.

In a reanalysis of two U.S. cohort studies, Krewski et al. (2000) found that persons with the lowest education experienced the largest health effects from air pollution. We found in the case–cohort analyses suggestive evidence for higher effects for BS in those with low education and in those with low fruit consumption. Low fruit consumption occurred significantly more in low education households, suggesting that the possible modifying effect of education on air pollution estimates may result from differences in fruit consumption between subjects with different educational levels. Fruit consumption may protect against oxidative stress, which is one of the main pathways from air pollution to health effects (Kelly 2004). However, this is but one of a series of differences in potential risk factors that are related to educational level (e.g., physical activity patterns or other dietary habits).

Although not statistically significantly different, the effect estimates for natural-cause and lung cancer mortality were higher among never smokers, whereas the effect estimates for respiratory mortality were higher among current smokers. We do not have an explanation for this. Stronger associations between air pollution and lung cancer mortality in never-smokers have been observed in the large ACS study as well (Pope et al. 2002), whereas for respiratory mortality the RRs for never-, ex-, and current smokers were all close to unity (Pope et al. 2004).

For subjects who did not move during the follow-up period, higher effect estimates were found for the BS overall concentrations than in all subjects. Such results could be related to more accurate exposure assessment for subjects who did not move. Because moving is a time-dependent variable, a limitation of this analysis is that we restricted the analysis only to the subjects who did not move during the follow-up period, ignoring the person-years that subjects lived at their baseline address before they moved. This may have created bias (Pearce 1992). The association between air pollution and mortality will still probably not differ for subjects who did not move and for subjects living at their baseline address before they moved.

Background concentrations showed a decrease over time (Beelen et al. 2007), but the correlations between the periods 1976–1985 and 1987–1996 were high (> 0.9). Therefore it was difficult to evaluate which time period was most important in relation to health effects. The spatial correlation between different pollutants was also high (Beelen et al. 2007). This makes it very difficult to isolate the health effects of individual pollutants because they act as indicators of a mixture of air pollutants coming from the same sources (Kjellstrom et al. 2002).

We used traffic intensity data only for the year 1986. Traffic intensities increased over time, but the correlations between traffic intensities from 1986 and traffic intensities from the years during follow-up were all > 0.92, supporting the use of traffic intensities from one year to represent a long-term average (Beelen et al. 2007). However, the high correlations made it difficult to assess which traffic intensity years are most important in relation to adverse health effects.

The results may not necessarily apply to current situations because emissions per vehicle have decreased in the last decades due to technical innovations and use of catalytic converters. However, the number of vehicles has increased in the same time period, and important pollutants such as NO_2_, ultrafine particles, and (diesel) soot are still being emitted.

A limitation of the exposure assessment method is that we assessed only outdoor concentrations at the baseline residential address, not taking into account factors related to infiltration of outdoor air pollution into the home such as air exchange rate. The questionnaire did not contain information about the work address. However, approximately 85% of the population had no paid job at baseline. Further, we had no information about the time participants spent at home or about the time commuting in traffic. The resulting misclassification is however likely to be nondifferential.

In conclusion, we found that traffic-related air pollution and several traffic exposure variables were associated with mortality in the full cohort. Relative risks were generally small. Associations between natural-cause and respiratory mortality were statistically significant for NO_2_ and BS. These findings add to the evidence found in studies on the health effects of long-term exposure to air pollution concentrations. Although the relative risks were small, the public health impact of exposure to air pollution may be substantial because the exposed population is large.

## Figures and Tables

**Table 1:** Number of deaths during follow-up.

Cause	ICD-9 codes	ICD-10 codes	No. of deaths
All cause	All	All	17,610
Natural cause	< 800	< V01	17,286
Cardiopulmonary	400–440 or 460–519	I10–I70 or J00–J99	7,153
Cardiovascular	400–440	I10–I70	6,137
Respiratory	460–519	J00–J99	1,016
Lung cancer	162	C33–C34	1,888
Other than cardiopulmonary or lung cancer	Not 400–440, not 162, and not 460–519, and < 800	Not I00–I70, not J00–J99, not C33–C34, and < V01	8,569

**Table 2:** Descriptive characteristics of subjects who died and who were alive at end of follow-up in the full cohort [among subjects for which geographic coordinates of the home address were available (*n* = 117,528)].

Characteristic	Cases (*n* = 17,286)	Noncases (*n* = 100,242)
Sex (men)	11,317 (65.5)	45,484 (45.4)
Age (years)	64 (60–67)	61 (58–65)
Cigarette-smoking status		
Never	4,788 (29.8)	40,113 (42.5)
Ex	5,063 (31.5)	29,899 (31.7)
Current	6,207 (38.7)	24,325 (25.8)
Cigar-smoking status		
Never	13,663 (82.7)	84,935 (88.3)
Ex	1,429 (8.6)	6,394 (6.7)
Current	1,438 (8.7)	4,844 (5.0)
Pipe-smoking status		
Never	15,227 (91.5)	90,351 (93.6)
Ex	865 (5.2)	4,200 (4.4)
Current	552 (3.3)	1,947 (2.0)
Percent of persons with low income in neighborhood	41 (36–47)	41 (36–46)
Percent of persons with high income in neighborhood	18 (12–24)	19 (13–25)
Percent of persons with low income in a COROP area	41 (36–45)	41 (36–45)
Percent of persons with high income in a COROP area	19 (18–23)	19 (18–23)

Values are number (%) or median (interquartile range).

**Table 3:** Adjusted RRs (95% CIs) for the association between exposure to BS, PM_2.5_, NO_2_, and SO_2_ (1987–1996) with cause-specific mortality in full cohort and case–cohort analyses (increment used to calculate RR).^a^

	No. of cases^b^		BS (10 μg/m^3^)		PM_2.5_ (10 μg/m^3^)		NO_2_ (30 μg/m^3^)		SO_2_ (20 μg/m^3^)	
Mortality	Full cohort	Case cohort	Full cohort	Case cohort	Full cohort	Case cohort	Full cohort	Case cohort	Full cohort	Case cohort
Natural cause	15,287	10,094	1.05 (1.00–1.11)	0.97 (0.83–1.13)	1.06 (0.97–1.16)	0.86 (0.66–1.13)	1.08 (1.00–1.16)	0.87 (0.69–1.10)	0.97 (0.90–1.05)	0.91 (0.71–1.16)
Cardiovascular	5,397	3,608	1.04 (0.95–1.13)	0.98 (0.81–1.18)	1.04 (0.90–1.21)	0.83 (0.60–1.15)	1.07 (0.94–1.21)	0.88 (0.66–1.17)	0.94 (0.82–1.06)	0.88 (0.65–1.18)
Respiratory	904	574	1.22 (0.99–1.50)	1.29 (0.91–1.83)	1.07 (0.75–1.52)	1.02 (0.56–1.88)	1.37 (1.00–1.87)	1.26 (0.74–2.15)	0.88 (0.64–1.22)	0.88 (0.51–1.50)
Lung cancer	1,670	1,059	1.03 (0.88–1.20)	1.03 (0.77–1.38)	1.06 (0.82–1.38)	0.87 (0.52–1.47)	0.91 (0.72–1.15)	0.80 (0.52–1.23)	1.00 (0.79–1.26)	0.99 (0.62–1.58)
Other	7,603	5,036	1.04 (0.97–1.12)	0.91 (0.78–1.07)	1.08 (0.96–1.23)	0.85 (0.65–1.12)	1.09 (0.98–1.21)	0.83 (0.66–1.06)	1.00 (0.90–1.12)	0.93 (0.72–1.19)

a Full cohort analyses adjusted for age, sex, smoking status, and area level indicators of socioeconomic status. Case–cohort analyses adjusted for age, sex, BMI, active smoking, passive smoking, education, occupational exposure, marital status, alcohol use, vegetable intake, fruit intake, energy intake, fatty acids intake, folate intake, fish consumption, and area-level indicators of socioeconomic status. BS, PM_2.5_, and NO_2_ are quantitative overall concentrations. SO_2_ is background concentration (including traffic intensity on nearest road in model). Number of person-years in full cohort analyses is 984,589, and number of person-years in case–cohort analyses is 28,522.

b The number of cases between full cohort and case–cohort adjusted analyses differs because the larger confounder model in the case–cohort analyses produces a higher number of subjects not available for analysis due to missing values.

**Table 4:** Adjusted RRs (95% CIs) for the association between traffic variables with cause-specific mortality in full cohort and case–cohort analyses.^a^

Exposure model	Full cohort	Case cohort
Natural-cause mortality		
Traffic intensity on nearest road	1.03 (1.00–1.08)	0.99 (0.88–1.11)
Traffic intensity in a 100-m buffer	1.02 (0.97–1.07)	0.98 (0.85–1.13)
Living near a major road	1.05 (0.97–1.12)	0.92 (0.74–1.15)
Cardiovascular mortality		
Traffic intensity on nearest road	1.05 (0.99–1.12)	1.03 (0.90–1.17)
Traffic intensity in a 100-m buffer	1.00 (0.92–1.08)	0.98 (0.82–1.16)
Living near a major road	1.05 (0.93–1.18)	0.93 (0.72–1.21)
Respiratory mortality		
Traffic intensity on nearest road	1.10 (0.95–1.26)	0.94 (0.71–1.25)
Traffic intensity in a 100-m buffer	1.21 (1.02–1.44)	1.23 (0.89–1.68)
Living near a major road	1.19 (0.91–1.56)	0.85 (0.50–1.43)
Lung cancer mortality		
Traffic intensity on nearest road	1.07 (0.96–1.19)	1.03 (0.87–1.22)
Traffic intensity in a 100-m buffer	1.07 (0.93–1.23)	1.10 (0.85–1.43)
Living near a major road	1.20 (0.98–1.47)	1.07 (0.70–1.64)
Other mortality		
Traffic intensity on nearest road	1.00 (0.94–1.06)	0.93 (0.82–1.06)
Traffic intensity in a 100-m buffer	0.99 (0.93–1.06)	0.93 (0.80–1.07)
Living near a major road	0.98 (0.88–1.09)	0.85 (0.68–1.07)

The number of person-years and number of cases for the full cohort and case–cohort analyses are shown in Table 3.

a The used confounders for the full cohort and case–cohort analyses are described in Table 3. RRs were calculated for differences from the 5th to the 95th percentile: for the traffic intensity on the nearest road: 10,000 mvh/24hr; for the traffic intensity in a 100-m buffer: 335,000 mvh/100m. RRs for living near a major road were calculated with reference category “not living near a major road.” All models included BS background concentration (1987–1996) as background concentration.
